# Pulmonary Function and Long-Term Respiratory Symptoms in Children and Adolescents After COVID-19

**DOI:** 10.3389/fped.2022.851008

**Published:** 2022-04-25

**Authors:** Leona Knoke, Anne Schlegtendal, Christoph Maier, Lynn Eitner, Thomas Lücke, Folke Brinkmann

**Affiliations:** University Children’s Hospital, Ruhr University Bochum, Bochum, Germany

**Keywords:** COVID-19, SARS-CoV-2, pulmonary function, dyspnea, children, adolescents, LCI

## Abstract

**Background:**

Persistent respiratory symptoms after severe acute respiratory syndrome coronavirus 2 (SARS-CoV-2) in adults are frequent, and there can be long-term impairment of pulmonary function. To date, only preliminary evidence is available on persistent respiratory sequelae of SARS-CoV-2 in children and adolescents. Our objective was to examine the long-term effects of symptomatic and asymptomatic SARS-CoV-2 infections on pulmonary function in this age group in a single-center, controlled, prospective study.

**Methods:**

Participants with serological or polymerase chain reaction-based evidence of SARS-CoV-2 infection were recruited from a population-based study of seroconversion rates. Multiple-breath washout (MBW), body plethysmography, and diffusion capacity testing were performed for children and adolescents. Participants were interviewed about their symptoms during the acute phase of infection and long-lasting symptoms. Cases were compared with SARS-CoV-2 seronegative controls from the same population-based study with and without history of respiratory infection within 6 months prior to assessment. Primary endpoints were differences in pulmonary function, including diffusion capacity and MBW, between participants with and without evidence of SARS-CoV-2 infection. Secondary endpoints included correlation between lung function and long-lasting symptoms as well as disease severity.

**Findings:**

In total, 73 seropositive children and adolescents (5–18 years) were recruited after an average of 2.6 months (range 0.4–6.0) following SARS-CoV-2 infection. Among 19 patients (27.1%) who complained of persistent or newly emerged symptoms since SARS-CoV-2, 8 (11.4%) reported respiratory symptoms. No significant differences were detected in frequency of abnormal pulmonary function when comparing cases with 45 controls, including 14 (31.1%) with a history of previous infection (SARS-CoV-2: 12, 16.4%; controls: 12, 27.7%; odds ratio 0.54, 95% confidence interval 0.22–1.34). Only two patients with persistent respiratory symptoms showed abnormal pulmonary function. Multivariate analysis revealed reduced forced vital capacity (*p* = 0.012) in patients with severe SARS-CoV-2 infection.

**Interpretation:**

Pulmonary function is rarely impaired in children and adolescents after SARS-CoV-2 infection, except from those with severe infection, and did not differ between SARS-CoV-2 and other previous infections, suggesting that SARS-CoV-2 is not more likely to cause pulmonary sequelae than other infections. The discrepancy between persisting respiratory symptoms and normal pulmonary function suggests a different underlying pathology such as dysfunctional breathing.

## Introduction

The acute clinical course of children and adolescents with severe acute respiratory syndrome coronavirus 2 (SARS-CoV-2) infection is typically less severe than that of adults (1). However, there lies a proportion of adult and pediatric patients with long-lasting symptoms after symptomatic or asymptomatic acute infection. Prolonged coronavirus disease 2019 (COVID-19) consists of all symptoms that persist or newly emerge from 4 to >12 weeks after acute SARS-CoV-2 infection (2).

In one study, up to 87% of adults hospitalized owing to COVID-19 reported persistent symptoms (e.g., fatigue, dyspnea, cough, and gastrointestinal symptoms) approximately 2 months after SARS-CoV-2 infection (3). In another cohort of inpatients and outpatients in the same age range, only 4.5% remained symptomatic, with similar symptoms 8 weeks after COVID-19 (1). There is only preliminary evidence of persistent symptoms in children. Buonsenso et al. reported that 52.7% of their study population had at least one persistent symptom ≥ 4 months after SARS-CoV-2 infection, while 14.7% stated that they had persistent respiratory issues (4).

Long-lasting deterioration of pulmonary function has been described in adults (5–7). Lerum et al. reported that approximately 10% of adults hospitalized with COVID-19 had reduced forced vital capacity (FVC) and forced expiratory volume in one second (FEV_1_), and 24% showed significantly reduced diffusion capacity at a 3-month follow-up visit (5). However, there are very few reports in the literature on the frequency and extent of long-term changes in pulmonary function in children and adolescents following SARS-CoV-2 infection. Bottino et al. performed spirometry and diffusion capacity testing in seven children approximately 2 months after recovery from mild SARS-CoV-2 infection and did not detect any abnormalities (8). Lefkin Dobkin et al. came to a similar conclusion in their study, finding primarily normal results on body plethysmography and diffusion capacity testing for 15 children with ongoing respiratory symptoms approximately 3 months after COVID-19 (9). A case report described ventilation-perfusion inhomogeneities as possible causes of dyspnea in children and adolescents after COVID-19 (10). In a cohort of four pediatric patients with pulmonary hypertension, Morales-Demori et al. observed a mild decrease in lung function in three patients following SARS-CoV-2 infection (11). An Italian study examined the cardiopulmonary function of 115 athletes, most who were adolescents, after mild or asymptomatic SARS-CoV-2 infection before resuming sports activities and found slightly better FVC in mildly symptomatic rather than asymptomatic participants (12).

Evaluating the impact of respiratory sequelae in children is important since most children worldwide are at risk of infection with SARS-CoV-2. Also, studies in this field may be informative regarding the current vaccine guidelines discussion for children without underlying medical conditions.

We analyzed pulmonary function, including multiple-breath washout and diffusion capacity testing, in children and adolescents after COVID-19 and compared their results with those of a group of controls after excluding cases of SARS-CoV-2 infection. The long-term effects of COVID-19 are compared to the effects of other infections in children and adolescents.

## Patients and Methods

### Study Design and Population

We conducted a single-center, cross-sectional, prospective study to assess pulmonary function after COVID-19 in children and adolescents aged 5–18 years. This study was part of a population-based study at the University Children’s Hospital of Bochum, Germany; it assessed the rates of SARS-CoV-2 seroconversion in children and adolescents in Western Germany (CorKid). In the CorKid study, children and adolescents were tested for antibodies against SARS-CoV-2 during their regular check-up visits with their pediatrician.

We included participants with either polymerase chain reaction (PCR) and/or antibody-confirmed SARS-CoV-2 infections from August 2020 to March 2021. We recruited seropositive participants from the CorKid study and PCR-positive in- and outpatients tested at the University Children’s Hospital of Bochum or from outpatient care in the region. Regarding the control group, children aged 5–18 years with negative antibodies for SARS-CoV-2 and no other evidence of SARS-CoV-2 infection were recruited from the CorKid study. Exclusion criteria included inability to undergo lung function testing and missing written consent.

The participants and/or their guardians answered questionnaires regarding their medical history, especially acute and chronic pulmonary diseases, as well as current and previous medications. Any infections within 6 months prior to the assessment and their date, duration, and symptoms, as well as PCR test results for SARS-CoV-2, were recorded. We also documented persistent symptoms after COVID-19 in the COVID-19 group.

Based on these data, we subdivided the COVID-19 group into symptomatic and asymptomatic patients and cases that showed signs of respiratory tract infections (if cough, rhinitis, sore throat and/or dyspnea was confirmed) and other types of cases. The severity of infection was sub-classified as severe or non-severe. An infection was considered severe if it included dyspnea, fever >38.5°C for ≥ 5 days, bronchitis or pneumonia, and/or hospitalization for ≥ 1 day. Patients were classified as having chronic lung disease if they were diagnosed with pulmonary disease, such as bronchial asthma, or if they had one of the following: recurrent wheezing, pneumonia in the year before measurement, previous or current use of inhaled steroids for at least 4 weeks or long-lasting productive cough (≥ 8 weeks). We also divided the participants into two groups (follow-up 0–3 and 4–6 months), depending on the interval between infection and assessment.

### Pulmonary Function Testing

Body plethysmography and diffusing capacity testing for carbon monoxide (CO) were performed using MasterScreen Body/Diff (Vyaire, Hoechberg, Germany) based on the American Thoracic Society/European Respiratory Society guidelines (13, 14). We measured FEV_1_, FVC, mean expiratory flow at 75% (MEF_75_) and 25% (MEF_25_), as well as the diffusing capacity of the lung for carbon monoxide (DLCO) and diffusing capacity divided by the alveolar volume (DLCO/VA) standardized to *Z*-scores using global lung initiative reference values (15, 16). DLCO and DLCO/VA were adjusted for hemoglobin levels. N_2_ multiple-breath washout was measured using an Exhalyzer D (EcoMedics AG, Duernten, Switzerland) based on the current guidelines (17). We reported lung clearance index of 2.5% of the starting concentration (LCI_2.5%_), as recommended. All pulmonary function tests were perfomed by specially trained staff and assessed by two pediatric pneumologists blinded to patients’ SARS-CoV-2 status.

Pulmonary function was defined as abnormal if at least one measured parameter was pathological (LCI_2.5%_ > 7.9, FVC *Z*-score, FEV_1_ *Z*-score, MEF_75_ *Z*-score, MEF_25_ *Z*-score, DLCO *Z*-score, DLCO/VA *Z*-score < −1.96) or there was at least one borderline parameter (LCI_2.5%_ > 7.0, FVC *Z*-score, FEV_1_ *Z*-score, MEF_75_ *Z*-score, MEF_25_ *Z*-score, DLCO *Z*-score, DLCO/VA *Z*-score < –1 ≥ –1.96) for two different pulmonary function tests (N_2_ multiple-breath washout, body plethysmography, diffusion capacity).

Imaging of the lungs was performed only when clinically indicated.

### Statistics

The primary endpoints were differences in pulmonary function, including diffusion capacity and N_2_ multiple-breath washout between children and adolescents with and without evidence of SARS-CoV-2 infection. The secondary endpoints included the correlation between long-lasting symptoms, disease severity, and pulmonary function. Outcome variables included lung function parameters and symptoms at the time of infection and at the time of measurement. The time between infection and lung function assessment, severity of infection, underlying medical conditions, and other infections were considered potential confounders.

Descriptive statistics: We calculated the mean, median, range and standard deviation for continuous variables. Categorical values are expressed as absolute values and percentages.

Inferential statistics: Odds ratios (ORs) and 95% confidence intervals (95% CIs) were calculated for categorical values. Normal distribution was proven for all pulmonary function parameters using either the Kolmogorov–Smirnov test or the Levene test. An independent *t*-test or analysis of variance was used for between-group comparisons. A *post-hoc* analysis was performed using the least significant difference. All statistical analyses were performed using SPSS version 27 (SPSS for Windows; SPSS Inc., Chicago, IL, United States). Differences were considered statistically significant at *p* ≤ 0.05.

### Ethical Approval

The ethics committee of Ruhr-University, Bochum, Germany, approved the project (register number: 20-6927). All participants and/or their parents were informed of the study and provided written informed consent.

## Results

We enrolled 73 patients with confirmed SARS-CoV-2 infection, 26 patients (35.6%) were symptomatic during the acute phase. Among the 45 participants who served as controls, 14 (31.1%) had any symptomatic infection other than COVID-19 within 6 months before the assessment, probably and mostly mild viral infections. Excluding six COVID-19 patients without PCR results, the mean interval between infection and the date of testing was similar in both groups (COVID-19: 2.59 months [range 0.4–6.0]; controls: 3.43 months [range 1.03–6.3]). The epidemiological data were similar in both groups (Table 1).

**TABLE 1:** Epidemiological data.

	COVID-19 patients			Controls			OR (95% CI) all COVID-19 vs all Controls^2^	OR (95% CI) COVID-19 symptomatic vs COVID-19 asymptomatic^2^	OR (95% CI) COVID-19 symptomatic vs Controls with any other infection ^2^
	All	Symptomatic infection within the past 6 months	Asymptomatic infection within the past 6 months	All	Any other infection within the past 6 months	No infection within the past 6 months			
N	73	27*	46	45	14	31			
Sex, female	38 (52.0)	14 (51.9)	24 (52.2)	28 (62.2)	11 (78.6)	17 (54.8)	0.66 (0.31–1.41)	0.99 (0.38–2.56)	0.29 (0.07–1.29)
Age, years, *mean* ± *SD*	10.8 (± 3.3)	10.5 (± 3.2)	11.0 (± 3.3)	10 (± 3.5)	9.1 (± 3.4)	10.4 (± 3.5)			
Age group, 5–8 years	21 (28.8)	7 (25.9)	14 (30.4)	18 (40)	8 (57.1)	10 (32.3)	0.61 (0.28–1.32)	0.8 (0.28–2.32)	0.26 (0.07–1.03)
Age group, 9–18 years	52 (71.23)	19 (73.08)	33 (70.01)	27 (60.0)	6 (42.86)	21 (67.74)	1.65 (0.75–3.61)	1.25 (0.43–3.63)	3.81 (0.97–14.91)
Premature birth	4 (5.6)^1^	2 (8)^1^	2 (4.4)	5 (11.1)	2 (14.3)	3 (9.7)	0.48 (0.12–1.88)	1.76 (0.23–13.27)	0.48 (0.06–3.83)
History of admission to neonatal intensive care unit	13 (18.3)^1^	8 (32)^1^	5 (10.9)	4 (8.9)	2 (14.3)	2 (6.5)	2.3 (0.7–7.55)	3.45 (1–11.96)	2.53 (0.46–13.96)
With oxygen supple- mentation	4 (5.6)^1^	2 (8)^1^	2 (4.4)	1 (2.2)	0	1 (3.2)	2.63 (0.28–24.29)	1.76 (0.23–13.27)	–
Pulmonary disease, previous or current	17 (23.3)	8 (29.6)	9 (19.6)	10 (22.2)	3 (21.4)	7 (22.6)	1.06 (0.44–2.58)	1.73 (0.58–5.21)	1.54 (0.34–7.06)

*SD, standard deviation; OR, odds ratio; CI, confidence interval.*

*Data are expressed as n (%), unless specified otherwise.*

*One patient had a PCR-proven but asymptomatic SARS-CoV-2 infection and detectable antibodies against SARS-CoV-2 a few months after another symptomatic viral infection.

*^1^missing values n = 2; ^2^all p > 0.05.*

Disease severity and infection type did not differ significantly between the two groups. However, only 70.4% (*n* = 19) of children and adolescents with symptomatic SARS-CoV-2 infection showed respiratory symptoms, whereas 92.9% (*n* = 13) of the infections in the control group involved the respiratory tract. Rhinitis and cough were more frequent in the control group than in the COVID-19 group (rhinitis: OR 0.14, 95% CI 0.03–0.62; cough: OR 0.24, 95% CI 0.06–0.95). The other assessed symptoms did not show significant differences between the two groups (Table 2).

**TABLE 2:** Infection characteristics.

	COVID-19 patients	Controls	OR (95% CI)	*p*
			
	Symptomatic infection within the past 6 months	Any other infection within the past 6 months		
N	27*	14		
Respiratory tract infection	19 (70.4)	13 (92.9)	0.18 (0.02–1.64)	0.099
Severe infection	8 (29.6)	2 (14.3)	2.53 (0.46–13.96)	0.278
With dyspnea	3 (11.1)	1 (7.1)	1.63 (0.15–17.24)	0.685
With bronchitis or pneumonia	1 (3.7)	2 (14.3)	0.23 (0.02–2.8)	0.217
With fever > 38.5°C for > 5 days	3 (11.1)	0	–	0.195
With need for hospitalization	4 (14.8)	0	–	0.130
Symptoms				
Fever > 38.5°C	14 (51.9)	4 (28.6)	2.69 (0.67–10.74)	0.154
Rhinitis	9 (33.3)	11 (78.6)	0.14 (0.03–0.62)	**0.006**
Sore throat	12 (44.4)	9 (64.3)	0.44 (0.12–1.68)	0.228
Cough	10 (37)	10 (71.4)	0.24 (0.06–0.95)	**0.037**
Dyspnea	3 (11.1)	1 (7.1)	1.63 (0.15–17.24)	0.685
Headache	12 (44.4)	5 (35.7)	1.44 (0.38–5.45)	0.591
Limb pain	11 (40.7)	5 (35.7)	1.24 (0.33–4.71)	0.754
Fatigue	23 (85.2)	9 (64.3)	3.19 (0.7–14.66)	0.125
Diarrhea/Vomiting	4 (14.8)	2 (14.3)	1.04 (0.17–6.54)	0.964
Loss of smell/taste	5 (18.5)	0	–	0.086

*OR, odds ratio, CI confidence interval.*

*Data are expressed as n (%), unless specified otherwise.*

*One patient had a PCR-proven but asymptomatic SARS-CoV-2 infection and detectable antibodies against SARS-CoV-2 a few months after another symptomatic viral infection.

### Long-Term Complaints

Data on long-term complaints were only available for 70 participants of the COVID-19 group, 25 with symptomatic and 45 with asymptomatic SARS-CoV-2 infection. Nineteen (27.1%) children and adolescents of the COVID-19 group reported persistent or newly emerged symptoms after SARS-CoV-2 infection at the time of assessment. For 9 (47.3%) of them, the interval between acute SARS-CoV-2 infection and assessment was ≥ 12 weeks. The 19 patients were divided into 9 (47.3%) children and adolescents with symptomatic acute SARS-CoV-2 infection and 10 (52.6%) with an asymptomatic course of acute infection. 8 (42.1%) patients reported at least one respiratory symptom, of whom 6 (75.0%) had ongoing breathing problems and 2 (25%) had persistent cough (Table 3). The mean age of children and adolescents with long-term respiratory symptoms was similar to that of all patients with SARS-CoV-2 infection (all COVID-19: 10.8 ± 3.3, patients with long-term respiratory symptoms: 11.8 ± 3.9 [mean ± standard deviation in years]). In two patients, these respiratory problems emerged after asymptomatic SARS-CoV-2 infection. The frequency of long-term respiratory symptoms was significantly higher for children and adolescents with symptomatic COVID-19 than that with an asymptomatic course of infection (symptomatic COVID-19: 6, 24%; asymptomatic COVID-19: 2, 4.4%; OR 6.79, 95% CI 1.25–36.76). 10 (52.6%) patients experienced fatigue; 5 (26.3%) patients had both persistent respiratory symptoms and fatigue syndrome. Unfortunately, no data were available on the long-term complaints of children and adolescents from the control group with an infection within 6 months prior to assessment.

**TABLE 3:** Long-term complaints in children and adolescents with symptomatic and asymptomatic acute SARS-CoV-2 infection.

	Symptomatic acute SARS-CoV-2 infection	Asymptomatic acute SARS-CoV-2 infection	OR (95% CI)^1^
N	25	45	
Any long-term complaints	9 (36)	10 (22.2)	1.97 (0.67–5.78)
Cough	2 (8)	0	–
Breathing problems	4 (16)	2 (4.4)	4.1 (0.69–24.18)
Fatigue	5 (20)	5 (11.1)	2 (0.52–7.72)
Headache	1 (4)	2 (4.4)	0.9 (0.08–10.4)
Loss of smell/taste	4 (16)	3 (6.6)	2.67 (0.55–13.02)

*OR, odds ratio, CI confidence interval.*

*Data are expressed as n (%), unless specified otherwise.*

*^1^all p > 0.05.*

### Pulmonary Function Testing

Overall, 16.4% (*n* = 12) of the children and adolescents had abnormal pulmonary function after COVID-19 in relation to the 27.7% (*n* = 12) without SARS-CoV-2 infection (OR 0.54, 95% CI 0.22–1.34). 5 (41.7%) controls with abnormal pulmonary function had an infection within 6 months prior to assessment, and 4 (33.3%) had a preexisting pulmonary disease. In the COVID-19 group with abnormal pulmonary function, 4 (33.3%) had a symptomatic course of acute SARS-CoV-2 infection, 2 (16.7%) had a preexisting pulmonary disease. Comparing all participants with and without abnormal pulmonary function, no influence of SARS-CoV-2 status, previous or current pulmonary disease, symptomatic infection, or severity of infection was observed. Children and adolescents with symptomatic acute SARS-CoV-2 infection did not show abnormal pulmonary function testing more often than children and adolescents with other symptomatic infection within the last 6 months. Most cases of abnormal pulmonary function occurred in the 5- to 8-years age group (Table 4). This effect was mainly visible in the measurements of LCI_2.5%_ and FVC. Two of the eight patients reporting persistent respiratory complaints and three of the ten patients with fatigue syndrome showed abnormal pulmonary function.

**TABLE 4:** Characteristics of participants with and without abnormal^1^ pulmonary function.

	Abnormal pulmonary function	Normal pulmonary function	OR (95% CI)
N	24	94	
Positive SARS-CoV-2 status	12 (50)	61 (64.9)	0.54 (0.22–1.34)
Sex, female	10 (41.7)	56 (59.6)	0.48 (0.2–1.2)
Age, years, *mean* ± *SD*	8.6 (± 3.7)	11.0 (± 3.1)	
Age group, 5–8 years	17 (70.8)	22 (23.4)	7.95 (2.92–21.63)
Age group, 9–18 years	7 (29.2)	72 (76.6)	0.13 (0.05–0.34)
Premature birth	3 (12.5)	6 (6.4)	2.1 (0.48–9.07)
History of admission to neonatal intensive care unit	3 (12.5)	14 (14.9)	0.82 (0.21–3.11)
With oxygen supplementation	2 (8.3)	3 (3.2)	2.76 (0.43–17.52)
Pulmonary disease, previous or current	6 (25)	21 (22.3)	1.16 (0.41–3.29)
Infection within 6 months prior to assessment	9 (37.5)	32 (34)	1.16 (0.46–2.95)
With severe infection	4 (16.7)	6 (6.4)	2.93 (0.76–11.37)
With hospitalization	1 (4.2)	3 (3.2)	1.32 (0.13–13.27)

*SD, standard deviation; OR, odds ratio; CI, confidence interval.*

*Data are expressed as n (%), unless specified otherwise.*

*^1^Abnormal pulmonary function defined as at least one measured parameter being pathological (LCI_2.5%_ > 7.9, FVC Z-score, FEV_1_ Z-score, MEF_75_ Z-score, MEF_25_ Z-score, DLCO Z-score, DLCO/VA Z-score < −1.96) or at least one borderline parameter (LCI_2.5%_ > 7.0, FVC Z-score, FEV_1_ Z-score, MEF_75_ Z-score, MEF_25_ Z-score, DLCO Z-score, DLCO/VA Z-score < −1 ≥ −1.96) in two different pulmonary function tests (N_2_ multiple-breath washout, body plethysmography, diffusion capacity).*

*LCI_2.5%_, lung clearance index at 2.5% of the starting concentration; FVC, forced vital capacity; FEV_1_, forced expiratory volume in the first second; MEF, mean expiratory flow at different pulmonary volume levels (75, 25); DLCO, diffusing capacity of the lungs for carbon monoxide; VA, alveolar volume.*

**FIGURE 1:**
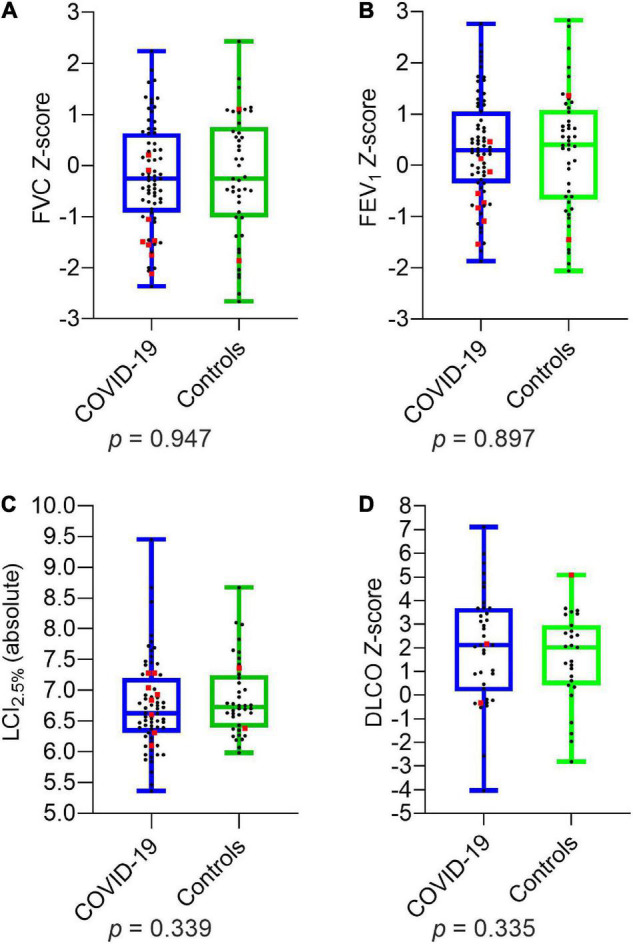
Depiction of pulmonary function parameters for COVID-19 and controls: **(A)** FVC *Z*-score **(B)** FEV_1_ *Z*-score **(C)** LCI_2.5%_ (absolute) **(D)** DLCO *Z*-score. Boxplots show medians, quartiles, minimum and maximum values. The dots represent the individual values of participants. Participants with severe infection within the last 6 months are marked as red squares. Differences were considered statistically significant at *p* ≤ 0.05. LCI_2.5%_, Lung Clearance Index at 2.5% of starting concentration; FVC, Forced Vital Capacity; FEV_1_, Forced Expiratory Volume in the first Second; DLCO, Diffusion Capacity of the lungs for Carbon Monoxide.

Comparing the two groups of children and adolescents with and without SARS-CoV-2 infection, there were no significant differences in LCI_2.5%_ (absolute) and age-related *Z*-scores of FVC, FEV_1_, MEF_75_, MEF_25_, DLCO, and DLCO/VA. The details are presented in Figure 1 and Table 5.

**TABLE 5:** Pulmonary function parameters.

	COVID-19 patients			Controls		
	All	Symptomatic infection within the past 6 months	Asymptomatic infection within the past 6 months	All	Any other infection within the past 6 months	No infection within the past 6 months
**Pulmonary function testing**						
N	73	27	46	45	14	31
Abnormal pulmonary function*	12 (16.44)	4 (14.81)	8 (17.39)	12 (26.67)	5 (35.71)	7 (22.58)
**N_2_ Multiple-breath washout**						
N	68	27	41	40	11	29
LCI_2.5_, absolute	6.75 ± 0.73	6.79 ± 0.62	6.72 ± 0.81	6.88 ± 0.61	6.79 ± 0.47	6.91 ± 0.66
Abnormal values	3 (4.4)	1 (3.7)	2 (4.9)	3 (7.5)	0	3 (10.3)
**Body plethysmography**						
N	71	26	45	45	14	31
FVC, *Z*-score	−0.21 ± 1.06	−0.26 ± 1.04	−0.19 ± 1.08	−0.21 ± 1.22	−0.25 ± 1.53	−0.19 ± 1.08
Abnormal values	5 (7)	1 (3.9)	4 (8.9)	5 (11.1)	2 (14.3)	3 (9.7)
FEV_1_, *Z*-score	0.3 ± 1.04	0.34 ± 1.04	0.28 ± 1.05	0.27 ± 1.19	0.2 ± 1.34	0.3 ± 1.13
Abnormal values	0	0	0	1 (2.2)	1 (7.1)	0
MEF_75_, *Z*-score	0.28 ± 1.1	−0.03 ± 1.13	0.46 ± 1.05	0.12 ± 1.09	−0.35 ± 1.38	0.34 ± 0.87
Abnormal values	3 (4.2)	2 (7.7)	1 (2.2)	1 (2.2)	1 (7.1)	0
MEF_25_, *Z*-score	0.86 ± 0.94	0.91 ± 0.68	0.83 ± 1.07	1.19 ± 1	1.04 ± 0.88	1.26 ± 1.06
Abnormal values	0	0	0	0	0	0
**Diffusion capacity**						
N	38	10	28	27	4	23
DLCO, *Z*-score	2.03 ± 2.35	2.56 ± 2.27	1.84 ± 2.39	1.55 ± 1.91	2.74 ± 2.7	1.35 ± 1.74
Abnormal values	2 (5.3)	0	2 (7.1)	1 (3.7)	0	1 (4.4)
DLCO/VA, *Z*-score	0.02 ± 0.76	−0.11 ± 1.09	0.07 ± 0.62	−0.21 ± 0.94	0.05 ± 1.39	−0.25 ± 0.88
Abnormal values	0	0	0	0	0	0

*LCI_2.5%_, lung clearance index at 2.5% of starting concentration; FVC, forced vital capacity; FEV_1_, forced expiratory volume in the first second; MEF, mean expiratory flow at different pulmonary volume levels (75, 25); DLCO, diffusing capacity of the lungs for carbon monoxide; VA, alveolar volume.*

*All pulmonary function parameters are presented as the mean ± standard deviation. Abnormal values were presented as n (%).*

*Abnormal pulmonary function defined as at least one measured parameter being pathological (LCI_2.5%_ > 7.9, FVC Z-score, FEV_1_ Z-score, MEF_75_ Z-score, MEF_25_ Z-score, DLCO Z-score, DLCO/VA Z-score < −1.96) or at least one borderline parameter (LCI_2.5%_ > 7.0, FVC Z-score, FEV_1_ Z-score, MEF_75_ Z-score, MEF_25_ Z-score, DLCO Z-score, DLCO/VA Z-score < −1 ≥ −1.96) in two different pulmonary function tests (N_2_ multiple-breath washout, body plethysmography, diffusion capacity).

The follow-up time (0–3 and 4–6 months) between infection and assessment date did not have any influence on pulmonary function parameters.

**FIGURE 2:**
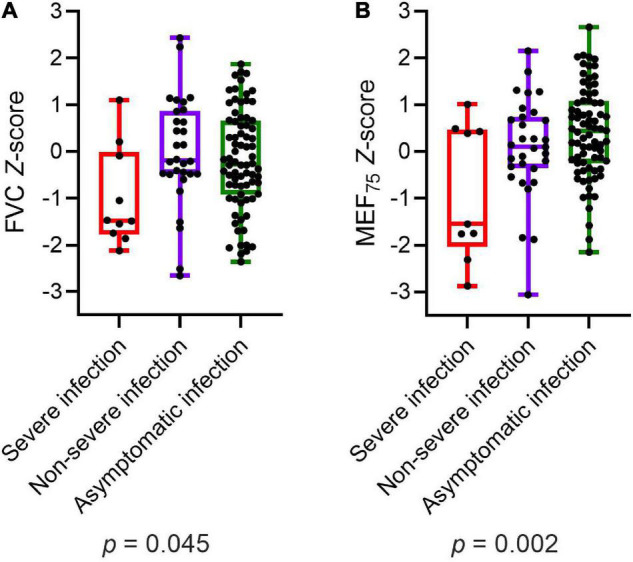
Depiction of **(A)** FVC *Z*-score and **(B)** MEF_75_ *Z*-score of all participants (COVID-19 and controls) divided into severe, non-severe and asymptomatic infection, respectively no infection in the control group. Boxplots show medians, quartiles, minimum and maximum values. The dots represent the individual values of participants. Differences were considered statistically significant at *p* ≤ 0.05. FVC, Forced Vital Capacity; MEF_75_, Mean Expiratory Flow at 75%.

Participants with severe infection within 6 months prior to the assessment had significantly lower values of FVC (*p* = 0.045) and MEF_75_ (*p* = 0.002) than those with non-severe infection and asymptomatic infection (Figure 2). MEF_25_, DLCO, DLCO/VA, and LCI_2.5%_ did not show any significant differences in *post-hoc* power analysis between children and adolescents with severe, non-severe or asymptomatic infection 6 months prior to the assessment. Within the COVID-19 group, similar results could be detected: patients with severe SARS-CoV-2 infection showed significantly lower values of FVC (*p* = 0.012), FEV_1_ (*p* = 0.014) and MEF_75_ (*p* = 0.028) compared to those with a non-severe symptomatic or asymptomatic infection. The details are presented in Table 6.

**TABLE 6:** *p-*values for analysis of variance.

	All participants (COVID-19 + Controls)			COVID-19		
Lung function parameter	Severe infection	Non-severe infection	Asymptomatic/no infection	Severe infection	Non-severe infection	Asymptomatic infection
FVC, *Z*-score	−0.92 ± 1.08	−0.06 ± 1.19	−0.19 ± 1.07	−1.08 ± 0.84	0.04 ± 0.95	−0.19 ± 1.08
	***p* = 0.045**			***p* = 0.012**		
FEV_1_, *Z*-score	−0.36 ± 0.93	0.48 ± 1.14	0.29 ± 1.08	−0.46 ± 0.67	0.63 ± 1.01	−0.36 ± 0.93
	*p* = 0.051			***p* = 0.014**		
MEF_75_, *Z*-score	−0.88 ± 1.45	0.08 ± 1.07	0.41 ± 0.98	−0.66 ± 1.59	0.21 ± 0.84	−0.88 ± 1.45
	***p* = 0.002**			***p* = 0.028**		
MEF_25_, *Z*-score	0.78 ± 0.65	1.01 ± 0.78	1.01 ± 1.08	0.92 ± 0.67	0.91 ± 0.7	0.78 ± 0.65
	*p* = 0.752			*p* = 0.941		
DLCO, *Z*-score	2.31 ± 2.71	2.7 ± 2.31	1.62 ± 2.12	0.92 ± 1.77	2.98 ± 2.29	1.84 ± 2.39
	*p* = 0.311			*p* = 0.394		
DLCO/VA, *Z*-score	0.41 ± 0.57	−0.2 ± 1.22	−0.08 ± 0.76	0.11 ± 0.32	−0.17 ± 1.22	0.07 ± 0.62
	*p* = 0.553			*p* = 0.748		
LCI_2.5_, absolute	6.76 ± 0.44	6.8 ± 0.61	6.8 ± 0.75	6.73 ± 0.42	6.81 ± 0.68	6.72 ± 0.81
	*p* = 0.977			*p* = 0.705		

*LCI_2.5%_, lung clearance index at 2.5% of starting concentration; FVC, forced vital capacity; FEV_1_, forced expiratory volume in the first second; MEF, mean expiratory flow at different pulmonary volume levels (75, 25); DLCO, diffusing capacity of the lungs for carbon monoxide; VA, alveolar volume.*

*All pulmonary function parameters are presented as the mean ± standard deviation.*

Only one child with pneumonia underwent pulmonary imaging during acute SARS-CoV-2 infection. No other child or adolescent required imaging of the lungs based on the medical assessment.

## Discussion

### Main Findings

To the best of our knowledge, our study is the first to compare pulmonary function in symptomatic and asymptomatic children and adolescents with and without evidence of SARS-CoV-2 infection; no difference between these two groups was observed. Respiratory symptoms did not correlate well with pulmonary function parameters. The only risk factor for any impairment of lung function was the severity of the acute infection.

### Acute Symptoms and Severity

Pulmonary involvement, including severe interstitial pneumonia and acute respiratory distress syndrome, in SARS-CoV-2 infection is the cause of high mortality and long-lasting morbidity in adults (7, 18). Severe pulmonary complications in children and adolescents are rare (19, 20), even in children with chronic lung diseases, such as cystic fibrosis (21). Several underlying mechanisms have been discussed to explain the differences in severity of pulmonary involvement between children and adults: although viral loads in children and adults during SARS-CoV-2 infection do not seem to differ a lot (22), invasion of respiratory cells and inflammatory response in the lower respiratory tract is more severe in adults. This may be caused by an increased expression of ACE receptors in respiratory tissue (23). In addition to this and maybe due to cross immunity after recent infection with other corona viruses and age dependent immunological variability, systemic and inflammatory response is increased in adults causing acute and permanent lung damage. Severity of SARS-CoV-2 infection is a predictor for persisting respiratory symptoms and lung function impairment (24). Therefore, the percentage of children and adolescents with persisting respiratory symptoms and lung function changes is much lower than in adults.

Most SARS-CoV-2 infections in our cohort were asymptomatic during the acute phase. Only 35.6% of the patients in our COVID-19 group stated that they had any symptoms during acute infection; respiratory symptoms were reported by 26%, and 11.1% described dyspnea with acute COVID-19. In an Italian cohort of children and adolescents (*n* = 16), 87% showed symptoms at the time of SARS-CoV-2 diagnosis, and 19% presented with respiratory symptoms (8). In addition, Funk et al. (20) found that most SARS-CoV-2 infections were symptomatic in the acute phase (94.7%); however, more than 70% had respiratory symptoms. The differences in our study are owing to the use of different cohorts, since our population also included children and adolescents in whom SARS-CoV-2 infection was detected incidentally during their routine check-ups.

### Long Term Symptoms

Long-term pulmonary symptoms and signs that persisted or emerged ≥ 4 weeks after acute infection, such as cough, dyspnea, or hyperreactive airways, were described by 11.4% of our patients. This is similar to the findings of an Italian cohort study, in which 14.7% of children had persistent respiratory problems for several months after SARS-CoV-2 infection (4). In adults, the frequency of persistent dyspnea 3 months after discharge from the hospital varies from 9 (25) to 54% (5). Persistent pulmonary issues after viral infections are a well-known phenomenon in children. For example, children with respiratory syncytial virus (RSV)-related bronchiolitis experience more wheezing episodes in the following 12 months (26), which can persist until the age of 6 years. Also, children with lower respiratory tract RSV infection in early childhood experience more frequent wheezing episodes (up to 4.3 times) than children without lower respiratory tract infections (27).

### Pulmonary Function in Adults

Pulmonary function in hospitalized adults is frequently impaired upon discharge. Mo et al. described that 47% of their patients had reduced DLCO, 25% had decreased total lung capacity (TLC), 9.1% had impaired FVC, and 13.6% showed reduced FEV_1_ (6). However, even 3–4 months after discharge, pulmonary function remains impaired in adults (5, 7, 28, 29). In a Norwegian cohort, FVC and FEV_1_ were reduced in approximately 10% and diffusion capacity in 24% of hospitalized adults 3 months after discharge (5). Qin et al. detected significant differences in DLCO levels between discharged patients with severe and non-severe COVID-19 at the 3-month follow-up (25). Lower DLCO and TLC were detected in patients with critical disease even after 6 months (28). A Mexican study investigating the correlation between persistent dyspnea and pulmonary function reported that patients with persistent dyspnea had significantly lower FVC, FEV_1_, and DLCO values than those without persistent dyspnea (30).

### Pulmonary Function in Children

As shown in our cohort, pulmonary function impairment in children after SARS-CoV-2 infection is rare. LCI_2.5%_ was abnormal in 4.4% of children and adolescents with COVID-19, 7% had reduced FVC, and 5.3% showed impaired DLCO. None of the patients had pathological FEV_1_ values after the SARS-CoV-2 infection. Some children in the control group (8/46) or after asymptomatic SARS-CoV-2 infection (7/31) showed reduced values in LCI_2.5%_ or FVC values. The majority of them (6/8 and 4/7) were young and performed their first lung function, so technical issues are probable, although the measurements were technically acceptable referring to international standards (13, 14, 17). Two children with reduced diffusion capacity were exposed to cigarette smoke. Therefore, we would not interpret these findings as results of SARS-CoV-2 infection. In total, no differences in the frequency of abnormal pulmonary function were observed between children and adolescents with symptomatic SARS-CoV-2 infection and other symptomatic infections. This supports the preliminary findings of the study by Bottino et al., in which seven children did not show any abnormalities in spirometry and diffusion capacity testing approximately 2 months after their mild SARS-CoV-2 infection (8). Our findings suggest that SARS-CoV-2 is not more likely than other infections to cause long-term sequelae in children and adolescents. Pulmonary function was normal in 75% of our participants, including children and adolescents with persistent respiratory symptoms. This finding is in line with the results of Lefkin Dobkin et al., who found primarily normal results on body plethysmography and diffusion capacity for 15 children with ongoing respiratory symptoms (9). In our cohort, LCI_2.5%_, FEV_1_, DLCO, and DLCO/VA had no significant differences between patients and controls. Minimal changes persisted for up to 6 months only in children and adolescents with more severe infections (SARS-CoV-2 or other infections). Similarly, other studies on children with viral infections, such as RSV or rhinovirus, detected acute and long-term loss of pulmonary function (26, 31, 32). Other known risk factors for reduced pulmonary capacity, such as preterm birth (33) or underlying pulmonary disease had no effect on our cohort.

### Strengths of the Study

The major strengths of our study are the prospective physiological multi-domain approach, which included body plethysmography, multiple-breath washout, and diffusion capacity testing as well as the inclusion of a control group.

### Limitations

Limitations of this single-center design with a rather small sample size include the heterogeneous population, patient-reported symptoms, and lack of information on pulmonary function before SARS-CoV-2 infection as well as long-term outcome of symptoms in controls. In addition, there was no information on specific pathogens of the infections within the control group owing to lack of testing in outpatient clinics. Moreover, age-dependent inconsistencies in pulmonary function performance within the youngest participants led to marginally pathologic values within the COVID-19 and control group. Furthermore, our cohort did not include children or adolescents with critical respiratory involvement during the acute COVID-19 pandemic.

## Conclusion

The proportion of children and adolescents with lung function impairment after symptomatic or asymptomatic SARS-CoV-2 infection was not significantly higher than that in children and adolescents without a recent history of any kind of infection or those with a non-SARS-CoV-2 infection in the past 6 months. Most patients with persistent respiratory symptoms do not have impaired lung capacity. The severity of infection was the only predictor of mild pulmonary function changes. In conclusion, our findings suggest that after SARS-CoV-2 infection, children and adolescents do not experience persistent deterioration of respiratory function, including body plethysmography, multiple-breath washout, and diffusion capacity testing.

### Outlook

Further studies with a larger, more representative cohort (including patients with critical respiratory involvement) performed over a prolonged period are needed to clarify long-term respiratory impairment after SARS-CoV-2 infection in children and adolescents. The discrepancy between subjective persistent respiratory complaints and normal pulmonary function may be caused by functional respiratory disorders, such as hyperventilation, as already described in adults (34, 35). Thus, further studies, including treadmill testing, would be useful and have already been initiated in our cohort.

## Data Availability Statement

The raw data supporting the conclusions of this article will be made available by the authors, without undue reservation.

## Ethics Statement

The studies involving human participants were reviewed and approved by the Ethics committee of the Ruhr University, Bochum, Germany (register number: 20-6927). Written informed consent to participate in this study was provided by the participants’ legal guardian/next of kin.

## Author Contributions

CM, TL, FB, and AS conceptualized the study design and protocol and supervised patient management. Founding was performed using TL, CM, and FB. LK, AS, and LE examined the patients and collected and curated the data. LK, AS, CM, and FB performed formal analyses. LK drafted the manuscript with contributions from the intellectual content of AS, LE, CM, TL, and FB. All authors have read and approved the final version of the manuscript.

## Conflict of Interest

The authors declare that the research was conducted in the absence of any commercial or financial relationships that could be construed as a potential conflict of interest.

## Publisher’s Note

All claims expressed in this article are solely those of the authors and do not necessarily represent those of their affiliated organizations, or those of the publisher, the editors and the reviewers. Any product that may be evaluated in this article, or claim that may be made by its manufacturer, is not guaranteed or endorsed by the publisher.

## References

[1] Sudre CH Murray B Varsavsky T Graham MS Penfold RS Bowyer RC Attributes and predictors of long COVID. *Nat Med.* (2021) 27:626–31. 10.1038/s41591-021-01292-y 33692530 PMC7611399

[2] National Institute for Health and Care Excellence [NICE]. *COVID-19 Rapid Guideline: Managing the Long-Term Effects of COVID-19.* London: National Institute for Health and Care Excellence (2020). 33555768

[3] Carfì A Bernabei R Landi F Gemelli Against COVID-19 Post-Acute Care Study Group. Persistent symptoms in patients after acute COVID-19. *JAMA.* (2020) 324:603–5. 10.1001/jama.2020.12603 32644129 PMC7349096

[4] Buonsenso D Munblit D De Rose C Sinatti D Ricchiuto A Carfi A Preliminary evidence on long COVID in children. *Acta Paediatr.* (2021) 110:2208–11. 10.1111/apa.15870 33835507 PMC8251440

[5] Lerum TV Aaløkken TM Brønstad E Aarli B Ikdahl E Lund KMA Dyspnoea, lung function and CT findings 3 months after hospital admission for COVID-19. *Eur Respir J.* (2021) 57:2003448. 10.1183/13993003.03448-2020 33303540 PMC7736755

[6] Mo X Jian W Su Z Chen M Peng H Peng P Abnormal pulmonary function in COVID-19 patients at time of hospital discharge. *Eur Respir J.* (2020) 55:2001217. 10.1183/13993003.01217-2020 32381497 PMC7236826

[7] Anastasio F Barbuto S Scarnecchia E Cosma P Fugagnoli A Rossi G Medium-term impact of COVID-19 on pulmonary function, functional capacity and quality of life. *Eur Respir J.* (2021) 58:2004015. 10.1183/13993003.04015-2020 33574080 PMC7877327

[8] Bottino I Patria MF Milani GP Agostoni C Marchisio P Lelii M Can asymptomatic or non-severe SARS-CoV-2 infection cause medium-term pulmonary sequelae in children? *Front Pediatr.* (2021) 9:621019. 10.3389/fped.2021.621019 34084763 PMC8168403

[9] Lefkin Dobkin SC Collaco JM McGrath-Morrow SA. Protracted respiratory findings in children post-SARS-CoV-2 infection. *Pediatr Pulmonol.* (2021) 56:3682–7. 10.1002/ppul.25671 34534416 PMC8662194

[10] Buonsenso D Di Giuda D Sigfrid L Pizzuto DA Di Sante G De Rose C Evidence of lung perfusion defects and ongoing inflammation in an adolescent with post-acute sequelae of SARS-CoV-2 infection. *Lancet Child Adolesc Health.* (2021) 5:677–80. 10.1016/S2352-4642(21)00196-6 34339624 PMC8324416

[11] Morales-Demori R Mallory GB Chartan C Coleman R Ruiz F Villafranco N Outcomes of COVID-19 infection in pediatric pulmonary hypertension: a single-center experience. *Pediatr Pulmonol.* (2021) 56:3960–5. 10.1002/ppul.25650 34460150 PMC8662244

[12] Chicco D Rispoli F De Nardi L Romano S Mazzolai M Bobbo M Cardio-pulmonary function among children with mild or asymptomatic COVID-19 infection needing certification for return-to-play. *J Paediatr Child Health.* (2021). 10.1111/jpc.15685 [Epub ahead of print]. 34396633 PMC8447300

[13] Graham BL Brusasco V Burgos F Cooper BG Jensen R Kendrick A 2017 ERS/ATS standards for single-breath carbon monoxide uptake in the lung. *Eur Respir J.* (2017) 49:1600016. 10.1183/13993003.00016-2016 28049168

[14] Miller MR Hankinson J Brusasco V Burgos F Casaburi R Coates A Standardisation of spirometry. *Eur Respir J.* (2005) 26:319–38. 10.1183/09031936.05.00034805 16055882

[15] Quanjer PH Stanojevic S Cole TJ Baur X Hall GL Culver BH Multi-ethnic reference values for spirometry for the 3–95-yr age range: the global lung function 2012 equations. *Eur Respir J.* (2012) 40:1324–43. 10.1183/09031936.00080312 22743675 PMC3786581

[16] Stanojevic S Graham BL Cooper BG Thompson BR Carter KW Francis RW Official ERS technical standards: global lung function initiative reference values for the carbon monoxide transfer factor for Caucasians. *Eur Respir J.* (2017) 50:1700010. 10.1183/13993003.00010-2017 28893868

[17] Robinson PD Latzin P Verbanck S Hall GL Horsley A Gappa M Consensus statement for inert gas washout measurement using multiple- and single- breath tests. *Eur Respir J.* (2013) 41:507–22. 10.1183/09031936.00069712 23397305

[18] Belli S Balbi B Prince I Cattaneo D Masocco F Zaccaria S Low physical functioning and impaired performance of activities of daily life in COVID-19 patients who survived hospitalisation. *Eur Respir J.* (2020) 56:2002096. 10.1183/13993003.02096-2020 32764112 PMC7411272

[19] Götzinger F Santiago-García B Noguera-Julián A Lanaspa M Lancella L Calò Carducci FI COVID-19 in children and adolescents in Europe: a multinational, multicentre cohort study. *Lancet Child Adolesc Health.* (2020) 4:653–61. 10.1016/S2352-4642(20)30177-2 32593339 PMC7316447

[20] Funk AL Florin TA Kuppermann N Tancredi DJ Xie J Kim K Outcomes of SARS-CoV-2-positive youths tested in emergency departments: the global PERN-COVID-19 study. *JAMA Netw Open.* (2022) 5:e2142322. 10.1001/jamanetworkopen.2021.42322 35015063 PMC8753506

[21] Bain R Cosgriff R Zampoli M Elbert A Burgel PR Carr SB Clinical characteristics of SARS-CoV-2 infection in children with cystic fibrosis: an international observational study. *J Cyst Fibros.* (2020) 20:25–30. 10.1016/j.jcf.2020.11.021 33309057 PMC7713571

[22] Baggio S L’Huillier AG Yerly S Bellon M Wagner N Rohr M Severe acute respiratory syndrome coronavirus 2 (SARS-CoV-2) viral load in the upper respiratory tract of children and adults with early acute coronavirus disease 2019 (COVID-19). *Clin Infect Dis.* (2021) 73:148–50. 10.1093/cid/ciaa1157 32761228 PMC7454380

[23] Bunyavanich S Do A Vicencio A. Nasal gene expression of angiotensin-converting enzyme 2 in children and adults. *JAMA.* (2020) 323:2427–9. 10.1001/jama.2020.8707 32432657 PMC7240631

[24] Lingappan K Karmouty-Quintana H Davies J Akkanti B Harting MT. Understanding the age divide in COVID-19: why are children overwhelmingly spared? *Am J Physiol Lung Cell Mol Physiol.* (2020) 319:L39–44. 10.1152/ajplung.00183.2020 32491949 PMC7324935

[25] Qin W Chen S Zhang Y Dong F Zhang Z Hu B Diffusion capacity abnormalities for carbon monoxide in patients with COVID-19 at three-month follow-up. *Eur Respir J.* (2021) 58:2003677. 10.1183/13993003.03677-2020 33574077 PMC7877322

[26] Cifuentes L Caussade S Villagrán C Darrigrande P Bedregal P Valdivia G Risk factors for recurrent wheezing following acute bronchiolitis: a 12-month follow-up. *Pediatr Pulmonol.* (2003) 36:316–21. 10.1002/ppul.10365 12950045

[27] Stein RT Sherrill D Morgan WJ Holberg CJ Halonen M Taussig LM Respiratory syncytial virus in early life and risk of wheeze and allergy by age 13 years. *Lancet.* (1999) 354:541–5. 10.1016/S0140-6736(98)10321-5 10470697

[28] Riou M Marcot C Oulehri W Enache I Pistea C Chatron E Respiratory follow-up after hospitalization for COVID-19: who and when? *Eur J Clin Invest.* (2021) 51:e13603. 10.1111/eci.13603 33998683 PMC8209926

[29] Bellan M Soddu D Balbo PE Baricich A Zeppegno P Avanzi GC Respiratory and psychophysical sequelae among patients with COVID-19 four months after hospital discharge. *JAMA Netw Open.* (2021) 4:e2036142. 10.1001/jamanetworkopen.2020.36142 33502487 PMC7841464

[30] Cortés-Telles A López-Romero S Figueroa-Hurtado E Pou-Aguilar YN Wong AW Milne KM Pulmonary function and functional capacity in COVID-19 survivors with persistent dyspnoea. *Respir Physiol Neurobiol.* (2021) 288:103644. 10.1016/j.resp.2021.103644 33647535 PMC7910142

[31] Kitcharoensakkul M Bacharier LB Schweiger TL Wilson B Goss CW Lew D Lung function trajectories and bronchial hyperresponsiveness during childhood following severe RSV bronchiolitis in infancy. *Pediatr Allergy Immunol.* (2021) 32:457–64. 10.1111/pai.13399 33098584 PMC8200049

[32] Guilbert TW Singh AM Danov Z Evans MD Jackson DJ Burton R Decreased lung function after preschool wheezing rhinovirus illnesses in children at risk to develop asthma. *J Allergy Clin Immunol.* (2011) 128:532–8.e10. 10.1016/j.jaci.2011.06.037 21878241 PMC3233203

[33] Sonnenschein-van der Voort AM Arends LR de Jongste JC Annesi-Maesano I Arshad SH Barros H Preterm birth, infant weight gain, and childhood asthma risk: a meta-analysis of 147,000 European children. *J Allergy Clin Immunol.* (2014) 133:1317–29. 10.1016/j.jaci.2013.12.1082 24529685 PMC4024198

[34] de Groot EP Duiverman EJ Brand PL. Dysfunctional breathing in children with asthma: a rare but relevant comorbidity. *Eur Respir J.* (2013) 41:1068–73. 10.1183/09031936.00130212 23018913

[35] Motiejunaite J Balagny P Arnoult F Mangin L Bancal C d’Ortho MP Hyperventilation: a possible explanation for long-lasting exercise intolerance in mild COVID-19 survivors? *Front Physiol.* (2021) 11:614590. 10.3389/fphys.2020.614590 33536937 PMC7849606

[36] Knoke L Schlegtendal A Maier C Eitner L Lücke T Brinkmann F. More complaints than findings – Long-term pulmonary function in children and adolescents after COVID-19. *medRxiv.* [Preprint]. (2021). 10.1101/2021.06.22.21259273 PMC9081758 35547532

